# Neighborhood Disadvantage, Individual Experiences of Racism, and Breast Cancer Survival

**DOI:** 10.1001/jamanetworkopen.2025.3807

**Published:** 2025-04-07

**Authors:** Etienne X. Holder, Mollie E. Barnard, Nuo N. Xu, Lauren E. Barber, Julie R. Palmer

**Affiliations:** 1 Slone Epidemiology Center at Boston University, Boston, Massachusetts; 2 Department of Medicine, Boston University Chobanian & Avedisian School of Medicine, Boston, Massachusetts; 3 Rollins School of Public Health, Emory University, Atlanta, Georgia

## Abstract

**Importance:**

Due to the legacy of structural racism in the United States, Black US residents are more likely to live in disadvantaged neighborhoods regardless of individual socioeconomic status. Black women are also more likely to die from their breast cancer.

**Objective:**

To investigate whether neighborhood disadvantage and individual experiences of racism are associated with breast cancer mortality among Black US women.

**Design, Setting, and Participants:**

Prospective follow-up study conducted within the Black Women’s Health Study (BWHS), a nationwide cohort study of 59 000 self-identified Black females enrolled in 1995 and followed up by biennial questionnaires. BWHS participants diagnosed with breast cancer at stages I to III and who survived 12 months after initial diagnosis were included (n = 2290). Deaths and cause of death were identified through linkage with the National Death Index.

**Exposures:**

Geocoded participant addresses were linked with US Census Bureau and American Community Survey data to create neighborhood-level indices reflecting relative disadvantage. Data on experiences of racism were obtained from questionnaires.

**Main Outcomes and Measures:**

Breast cancer–specific and all-cause mortality. Hazard ratios (HRs) and 95% CIs were estimated from multivariable Cox proportional hazards regression models.

**Results:**

There were 305 breast cancer–specific deaths among 2290 women with invasive breast cancer (mean [SD] age at diagnosis, 56.7 [10.9] years) who were followed up for a median (IQR) of 10.5 (5.6-16.1) years. The mortality rate was 14.26 (95% CI, 11.01-17.51) per 1000 person-years for those living in the most disadvantaged neighborhoods and 8.82 (95% CI, 6.55-11.09) per 1000 person-years for those living in the least disadvantaged neighborhoods (HR, 1.47; 95% CI, 1.02-2.12). HRs for women living in areas of extreme economic deprivation (vs privilege) and for women who reported experiences of racism in at least 2 institutional spheres (vs none) were also greater than 1, but not statistically significant (extreme economic deprivation vs privilege: HR, 1.19; 95% CI, 0.88-1.61; racism in 2 spheres vs none: HR, 1.28; 95% CI, 0.96-1.73).

**Conclusions and Relevance:**

In this cohort of Black women, living in disadvantaged neighborhoods was associated with a higher mortality from breast cancer, even after accounting for stage at diagnosis, treatments, and individual-level lifestyle factors. Community-level interventions to reduce environmental stressors and improve conditions in disadvantaged neighborhoods, including access to high-quality cancer care, may be critical to reducing racial disparities in breast cancer survival.

## Key Points

**Question:**

Do neighborhood-level factors and individual experiences of racism affect breast cancer mortality among Black women living in the United States?

**Findings:**

In this cohort study of 2290 Black women with breast cancer, living in neighborhoods of concentrated deprivation was associated with a statistically significant 47% greater risk of dying from breast cancer, even after accounting for stage at diagnosis, cancer treatment, and individual-level lifestyle factors. There was not evidence of an association of self-reported experiences of racism with breast cancer–specific mortality.

**Meaning:**

These findings suggest that community-level interventions may be critical to reducing racial disparities in breast cancer survival.

## Teaser

This cohort study evaluates whether neighborhood disadvantage and individual experiences of racism are associated with mortality among Black women with breast cancer who participated in Black Women’s Health Study.

## Introduction

Breast cancer is the leading cause of cancer death among US Black women, who are 41% more likely to die from their breast cancer than US White women.^1^ Racial disparities in breast cancer survival persist even when well-established prognostic factors are taken into account.^2,3^ It is critical that social and economic factors be considered.^4^

Neighborhood disadvantage is a likely contributor to disparities in breast cancer outcomes. The legacy of structural racism, including redlining mortgage practices, has resulted in Black US residents being more likely to live in disadvantaged and economically deprived neighborhoods, regardless of their individual socioeconomic (SES) status.^5^ Living in economically disadvantaged neighborhoods has been associated with excess weight gain,^6^ less access to and intake of fruits and vegetables,^7^ and higher allostatic load,^8^ which may impact breast cancer outcomes. Previous studies have generally found associations of indices of neighborhood SES with breast cancer mortality, but most research has been conducted within a specific state and results specific to Black women have been inconsistent.^9,10,11,12,13,14,15,16,17^

Interpersonal racism may also play a role in the disproportionately higher breast cancer mortality in US Black women. Experiences of racism may be detrimental to symptom assessment and act as a barrier to seeking or obtaining timely health care, independent of neighborhood-level factors.^18^ Chronic distress from experiencing racism may increase allostatic load^19,20^ and influence biologic aging, leading to a worse prognosis after breast cancer.^18,19,21^ In the current study, we investigated whether neighborhood disadvantage, economic segregation, and personal experiences of racism are associated with breast cancer–specific and all-cause mortality in a cohort of Black women with breast cancer from the Black Women’s Health Study (BWHS).

## Methods

The BWHS, established in 1995, is a nationwide prospective cohort study of 59 000 self-identified Black women.^22^ In brief, Black women, aged 21 through 69 years at baseline, enrolled in the study by completing a questionnaire mailed to subscribers of Essence magazine and members of several professional organizations. The baseline and biennial follow-up questionnaires ask about major health conditions, medical history, reproductive history, lifestyle, and many other factors. The study protocol was approved by the institutional review boards of the Boston University Medical Campus and of participating cancer registries. Informed consent was implied by the return of questionnaires. We followed the Strengthening the Reporting of Observational Studies in Epidemiology (STROBE) reporting guidelines.

### Breast Cancer Survival Cohort

Incident breast cancers were identified through self-report on biennial questionnaires, linkages with 29 state cancer registries (from states where >95% of the BWHS participants reside), and linkage with the National Death Index (NDI). There were 3213 participants diagnosed with a first invasive breast cancer from 1997 through 2019. Cases with missing data on stage (n = 525) were ineligible for this analysis, as were cases with no active follow-up (n = 15). We excluded cases with stage IV breast cancer at the time of diagnosis (n = 115), and those who died within 12 months of their breast cancer diagnosis (n = 42) or did not respond to any questionnaires after diagnosis (n = 226). There were 2290 breast cancer cases in the analytic cohort (eFigure 1 in Supplement 1).

Data on tumor characteristics were abstracted from hospital pathology reports or state cancer registry data by trained study staff and coded following Surveillance, Epidemiology, and End Results (SEER) program guidelines. Data on initial breast cancer treatments, including chemotherapy, radiation therapy, endocrine therapy, and surgery, were abstracted, when available, from hospital and cancer registry records. Treatment data were also obtained through self-report on a supplemental questionnaire sent to all participants who reported a breast cancer diagnosis.

### Neighborhood-Level Indices

We evaluated 3 indices: neighborhood concentrated disadvantage (nDIS),^23^ neighborhood SES (nSES),^24^ and the Index of Concentration at the Extremes (ICE).^25^ Geocodes for participant addresses were linked to year 2000 US Census data for 1997 through 2004 and to American Community Survey data for 2005 and later. Participant addresses and resulting geocodes were updated throughout follow-up. We based the assessment of neighborhood-level indices on the most recent address after breast cancer diagnosis. The nDIS score was based on a factor analysis of 6 variables representing the percentages of individuals below the poverty line, receiving public assistance, living in female-headed households, unemployed, under 18 years of age, and Black residents.^23^ The 6 variables were weighted, as detailed previously, to obtain a single nDIS score, with a higher score indicating greater disadvantage.^26^ Similar methods were used to generate an nSES score based on 6 variables: median household income; median housing value; percentage of households receiving interest, dividend, or net rental income; percentage of adults aged 25 years or older who completed college; percentage of employed persons aged 16 years or older; and percentage of 2-parent households.^24,26^ Higher nSES score represented residence in more affluent neighborhoods. We calculated ICE for income economic segregation following the formula from Massey et al^27^: *ICEι* = (*Aι* − *Pι*)/*Tι*, where *Aι* represents the number people in the most privileged category (ι), *Pι* represents the number of people in the most deprived category (ι), and *Tι* represents the total number of people with a documented income level in a given neighborhood (ι), based on census tract–level data.^25,28^ The ICE for income measure ranges from 1 (extreme economic privilege) to −1 (extreme economic disadvantage). nDIS and nSES were measured at the block group level, and ICE was measured at the census tract level. Cut points for nDIS and nSES quartiles and ICE tertiles were based on the distributions of these variables in the entire BWHS population (59 000 participants).

### Experiences With Racism

Participants were asked about experiences of racism on the 1997, 2009, and 2019 questionnaires.^29^ For daily experiences of racism, participants were asked how often they experienced the following due to their race: “receive poorer service than other people at restaurants or stores,” “people act as if they think you are not intelligent,” “people act as if they are afraid of you,” “people act as if they think you are dishonest,” and “people act as if they are better than you.” Responses ranged from 1 to 5, with 1 representing never and 5 representing almost every day. Values were summed to create a daily racism score ranging from 5 to 25, and the score was then categorized in quartiles, based on the score distribution in the full cohort. To assess experiences of racism in institutional spheres, we asked women to report if they had ever been treated unfairly in employment, housing, or by the police. Responses were used to derive a single, 3-category variable: no to all domains, yes to 1 domain, and yes to 2 or more domains. Racism exposure variables were selected from the closest questionnaire after breast cancer diagnosis.

### Covariates

Data were available, either from questionnaires, medical records, or cancer registry records, on the following potential confounders: age at diagnosis, stage at diagnosis, estrogen receptor (ER) status, tumor grade, educational attainment, body mass index (BMI), menopausal status, type 2 diabetes, region of residence, alcohol consumption, cigarette smoking status, hours of vigorous physical activity per week, and insurance status. Data on time-varying covariates (eg, BMI, type 2 diabetes, physical activity) were obtained from the first returned BWHS biennial questionnaire after the participant reported her breast cancer diagnosis. Covariates were selected a priori.

### Ascertainment of Survival Status

Vital status was determined through linkage with the NDI. The cause of death was extracted from NDI data and death certificates.^30^ Deaths were categorized as breast cancer–specific if the primary or underlying cause of death was listed as breast cancer.

### Statistical Analysis

We used Kaplan-Meier curves and log-rank tests to visualize survival curves by category of nDIS, ICE, and nSES for each tumor stage and to check for violations of the proportional hazards assumption. We used Cox proportional hazards regression models to estimate hazard ratios (HRs) and 95% CIs for the associations of neighborhood indices and racism variables with risk of breast cancer–specific and all-cause mortality. For trend tests, we modeled exposures as a continuous variable. Participants were followed up from 12 months after their breast cancer diagnosis until death or December 31, 2022, whichever came first.

We present results from 3 models. The first was adjusted for age at breast cancer diagnosis (<40, 40-49, 50-59, 60-69, and ≥70 years). The second was additionally adjusted for stage at diagnosis (I, II, and III) and ER status (positive, negative, and missing). The third was additionally adjusted for tumor grade (well, moderately, or poorly differentiated and missing), radiation treatment (yes or not indicated because participant had a mastectomy, no, and unknown), endocrine therapy (yes or not indicated due to receptor status, no, and unknown), chemotherapy (yes, no, and unknown), and the following participant characteristics: educational attainment (≤12, 13-15, and ≥16 years); BMI, calculated as weight in kilograms divided by height in meters squared (<25, 25-30, 30-34, and ≥35), menopausal status (premenopausal, postmenopausal, and missing), type 2 diabetes (yes and no), region of residence (Northeast, South, Midwest, and West), alcohol consumption (<1, 1-6, and ≥7 drinks/week), cigarette smoking (current, past, and never), vigorous physical activity (0, <3, and ≥3 h/wk) and insurance status (yes, no, and missing). We investigated whether associations varied across strata of stage at diagnosis, ER status, age at diagnosis (<50 vs ≥50 years), and education level. *P* for heterogeneity was determined by comparing models with and without multiplicative interaction terms using likelihood ratio tests. For variables with substantial missing data (ER status, treatments, tumor grade, and menopause status) we included a missing indicator.^31^ For variables with nearly complete data (<3% missing), missing values were assigned to the most common category.

We conducted 2 post hoc analyses to better understand the observed association of nDIS with breast cancer mortality. First, we examined associations between each domain of the nDIS score and breast cancer mortality. Second, we cross-classified nDIS and daily experiences of racism and evaluated levels of that new variable in relation to breast cancer mortality.

Tests of statistical significance were 2-sided with α = .05. All analyses were performed using SAS version 9.4 (SAS Institute).

## Results

Among 2290 Black women (mean [SD] age at diagnosis, 56.7 [10.9] years) with invasive breast cancer, 602 deaths occurred, of which 305 were from breast cancer. As shown in Table 1, 1204 cancers (52.6%) were diagnosed at stage I, 843 (36.8%) at stage II, and 243 (10.6%) at stage III. ER status was available for 2069 cases (90.3%), among which 1422 (62.0%) were ER positive and 647 (28.3%) ER negative. Radiation treatment following lumpectomy was reported for 1074 women (86.4%) who had a lumpectomy only. Endocrine therapy was reported for 783 cases (55.1%) diagnosed with ER-positive cancer. The median (IQR) follow-up time was 10.5 (5.6-16.1) years.

**Table 1.** Tumor Characteristics, Treatments, and Participant Characteristics at Diagnosis

Characteristic	Participants, No. (%) (N = 2290)
Stage at diagnosis	
I	1204 (52.6)
II	843 (36.8)
III	243 (10.6)
Estrogen receptor status	
Positive	1422 (62.0)
Negative	647 (28.3)
Missing	221 (9.7)
Tumor grade at diagnosis	
Well differentiated	329 (14.4)
Moderately differentiated	807 (35.2)
Poorly differentiated	953 (41.6)
Missing	201 (8.8)
Treatments	
Radiation following lumpectomy	
Yes	928 (40.5)
No	146 (6.4)
Unknown	432 (18.9)
Mastectomy^a^	784 (34.2)
Endocrine therapy following ER-positive cancer	
Yes	783 (55.1)
No	313 (22.0)
Unknown	326 (22.9)
ER-negative/PR-negative cancer^b^	613 (26.8)
Chemotherapy	
Yes	1110 (48.5)
No	968 (42.3)
Unknown	212 (9.2)
Participant characteristics	
Age at diagnosis, y	
<40	113 (4.9)
40-49	520 (22.7)
50-59	735 (32.1)
60-69	631 (27.6)
≥70	291 (12.7)
BMI	
<25	494 (21.6)
25-29	785 (34.3)
30-34	565 (24.6)
≥35	446 (19.5)
Postmenopausal	1771 (77.3)
Educational attainment, y	
≤12	341 (15.0)
13-15	642 (28.0)
≥16	1307 (57.0)
Health insurance	
Yes	2066 (90.2)
No	121 (5.3)
Missing	103 (4.5)
Cigarette smoking, ever	901 (39.3)
Alcohol, drinks/wk	
<1	1662 (72.5)
1-6	538 (23.5)
≥7	90 (4.0)
Type 2 diabetes, ever	444 (19.4)
Vigorous physical activity, h/wk	
0	1156 (50.5)
<3	630 (27.5)
≥3	331 (14.5)
Missing	173 (7.5)
US region	
Northeast	543 (23.7)
South	812 (35.5)
Midwest	506 (22.1)
West	429 (18.7)

Abbreviations: BMI, body mass index (calculated as weight in kilograms divided by height in meters squared); ER, estrogen receptor; PR, progesterone receptor.

^a^ Radiation is not indicated after mastectomy.

^b^ Endocrine therapy is not indicated for ER-negative/PR-negative cancer.

In age-adjusted models, the HR for living in the most disadvantaged (nDIS quartile 4) relative to least disadvantaged (nDIS quartile 1) neighborhoods was 1.62 (95% CI, 1.15-2.29) (Table 2). The association was slightly attenuated with additional control for stage and ER status (HR, 1.57; 95% CI, 1.11-2.21), and was 1.47 (95% CI, 1.02, 2.12; *P* for trend = .04) in the fully adjusted model. For ICE, the multivariable HR for women living in extreme economic deprivation (ICE tertile 3) as compared with extreme economic privilege (ICE tertile 1) was 1.19 (95% CI, 0.88-1.61; *P* for trend = .05), but the findings were not statistically significant. Similarly, the HR for comparing low vs high nSES quartiles was not statistically significant (HR, 1.07; 95% CI, 0.74-1.55; *P* for trend = .21) (eTable 1 in Supplement 1).

**Table 2.** Associations Between Neighborhood-Level Indices and Breast Cancer Mortality in the Black Women’s Health Study, 1997 to 2022

Neighborhood-level indices	Breast cancer deaths, No.	Person-years, No.	HR (95% CI)		
			Age-adjusted^a^	Age, stage, ER status^b^	Fully adjusted^c^
Neighborhood concentrated disadvantage, quartile (n = 2126)					
First (least disadvantaged)	58	6574	1 [Reference]	1 [Reference]	1 [Reference]
Second	71	5923	1.35 (0.96-1.91)	1.35 (0.96-1.91)	1.33 (0.93-1.89)
Third	78	5841	1.54 (1.09-2.16)^d^	1.44 (1.02-2.02)^d^	1.38 (0.97-1.95)
Fourth (most disadvantaged)	74	5189	1.62 (1.15-2.29)^d^	1.57 (1.11-2.21)^d^	1.47 (1.02-2.12)^d^
* P* for trend	NA	NA	<.001	.004	.04
Index of Concentration at the Extremes (n = 2235)					
Extreme economic privilege	100	9122	1 [Reference]	1 [Reference]	1 [Reference]
Middle	99	8620	1.07 (0.81-1.41)	1.01 (0.77-1.34)	1.00 (0.75-1.32)
Extreme economic deprivation	100	7326	1.28 (0.97-1.69)	1.23 (0.93-1.63)	1.19 (0.88-1.61)
* P* for trend	NA	NA	.003	.008	.05

Abbreviations: ER, estrogen receptor; HR, hazard ratio; NA, not applicable.

^a^ HRs adjusted for age at diagnosis.

^b^ HRs additionally adjusted for stage at diagnosis and ER status.

^c^ HRs additionally adjusted for tumor grade, body mass index, radiation and endocrine therapy, chemotherapy, diabetes, menopause status, alcohol intake, cigarette smoking, vigorous physical activity, education, insurance status, and region.

^d^ *P* < .05.

Three of the 6 components of the nDIS index appeared to be the largest contributors to the observed association of nDIS with breast cancer mortality: percentage female-headed households, percentage unemployed, and percentage Black residents. HRs for 1-SD increases in each domain were 1.21 (95% CI, 1.08-1.35), 1.12 (95% CI, 1.01-1.24), and 1.11 (95% CI, 0.99-1.25), respectively (eTable 2 in Supplement 1).

Associations of nDIS with breast cancer–specific survival were generally consistent across strata of stage, ER status, age at diagnosis, and educational attainment (Table 3; eFigure 1 in Supplement 1). For ICE, while there was little evidence of an association with breast cancer mortality overall, a positive association was observed among women with ER-positive cancer (HR, 1.58; 95% CI, 1.04-2.39) and also among those diagnosed at stage II (HR, 1.51; 95% CI, 1.01-2.27) (Table 4; eFigure 2 in Supplement 1).

**Table 3.** Associations Between Neighborhood Concentrated Disadvantage and Breast Cancer Mortality, Within Strata of Age, Stage, ER Status, and Education Level at the Time of Breast Cancer Diagnosis^a^

Characteristics at diagnosis	Deaths, No.	HR (95% CI), by neighborhood concentrated disadvantage quartile (n = 2216)			
		First (least disadvantaged)	Second	Third	Fourth (most disadvantaged)
Stage					
I	68	1 [Reference]	1.71 (0.86-3.40)	1.14 (0.55-2.36)	1.90 (0.95-3.81)
II	137	1 [Reference]	1.31 (0.80-2.14)	1.37 (0.84-2.23)	1.39 (0.85-2.27)
III	76	1 [Reference]	1.11 (0.54-2.27)	1.71 (0.90-3.25)	1.73 (0.87-3.43)
* P* for heterogeneity	NA	.73			
ER status					
Positive	125	1 [Reference]	1.17 (0.70-1.97)	1.40 (0.84-2.34)	1.63 (1.00-2.66)^b^
Negative	110	1 [Reference]	2.35 (1.30-4.25)^b^	1.78 (0.97-3.25)	1.85 (0.98-3.48)
* P* for heterogeneity		.20			
Age at diagnosis, y^c^					
<50	101	1 [Reference]	1.45 (0.85-2.47)	1.21 (0.68-2.17)	1.31 (0.73-2.36)
≥50	180	1 [Reference]	1.23 (0.78-1.96)	1.57 (1.03-2.41)^b^	1.76 (1.15-2.71)^b^
* P* for heterogeneity		.43			
Education, y					
≤12	58	1 [Reference]	3.18 (1.16-8.72)^c^	1.82 (0.69-4.80)	2.33 (0.93-5.84)
13-15	79	1 [Reference]	1.12 (0.56-2.20)	1.13 (0.57-2.50)	1.52 (0.79-2.95)
≥16	144	1 [Reference]	1.24 (0.79-1.95)	1.44 (0.92-2.24)	1.29 (0.78-2.11)
* P* for heterogeneity	NA	.67			

Abbreviations: ER, estrogen receptor; HR, hazard ratio; NA, not applicable.

^a^ HRs adjusted for age at diagnosis, stage at diagnosis, and ER status.

^b^ *P* < .05.

^c^ Additionally adjusted for age at diagnosis continuously.

**Table 4.** Associations Between Index of Concentration at the Extremes and Breast Cancer Mortality, Within Strata of Age, Stage, ER Status, and Education Level at the Time of Breast Cancer Diagnosis^a^

Characteristics at diagnosis	Deaths, No.	HR (95% CI) by Index of Concentration at the Extremes (n = 2235)		
		Extreme economic privilege	Middle	Extreme economic deprivation
Stage				
I	76	1 [Reference]	0.90 (0.52-1.56)	0.92 (0.53-1.58)
II	143	1 [Reference]	1.13 (0.75-1.71)	1.51 (1.01-2.27)^b^
III	80	1 [Reference]	1.07 (0.63-1.83)	1.17 (0.67-2.06)
* P* for heterogeneity	NA	.66		
ER status				
Positive	133	1 [Reference]	0.96 (0.62-1.48)	1.58 (1.04-2.39)^b^
Negative	116	1 [Reference]	1.16 (0.74-1.82)	1.11 (0.71-1.76)
* P* for heterogeneity	NA	.40		
Age at diagnosis, y^c^				
<50	110	1 [Reference]	0.98 (0.62-1.54)	1.49 (0.95-2.34)
≥50	189	1 [Reference]	1.02 (0.72-1.46)	1.10 (0.77-1.57)
* P *for heterogeneity	NA	.43		
Education, y				
≤12	61	1 [Reference]	0.69 (0.34-1.41)	0.69 (0.35-1.35)
13-15	87	1 [Reference]	0.89 (0.53-1.51)	1.13 (0.67-1.92)
≥16	151	1 [Reference]	1.01 (0.69-1.47)	1.35 (0.91-2.01)
* P* for heterogeneity	NA	.51		

Abbreviations: ER, estrogen receptor; HR, hazard ratio; NA, not applicable.

^a^ HRs adjusted for age at diagnosis, stage at diagnosis, and ER status.

^b^ *P* < .05.

^c^ Additionally adjusted for age at diagnosis continuously.

Results from analyses of neighborhood variables and all-cause mortality are presented in eTable 3 in Supplement 1. All 3 variables were associated with all-cause mortality in analyses adjusted only for age, stage, and ER status, with HRs of 1.53 (95% CI, 1.21-1.93), 1.40 (95% CI, 1.14-1.70), and 1.41 (95% CI, 1.10-1.79) for the most extreme categories of nDIS, ICE, and nSES, respectively. However, with further control for factors, such as smoking, BMI, and alcohol intake, the HRs were attenuated and no longer statistically significant (nDIS: HR, 1.28; 95% CI, 1.00-1.64; ICE: HR, 1.21; 95% CI, 0.97-1.49; nSES: HR, 1.16; 95% CI, 0.90-1.51).

Multivariable-adjusted HRs were not statistically significant for women who reported the most frequent daily experiences of racism (quartile 4 vs 1); they were 1.11 (95% CI, 0.78-1.59) (Table 5) for breast cancer–specific mortality and 1.09 (95% CI, 0.85-1.42) for all-cause mortality (eTable 5 in Supplement 1). The multivariable-adjusted HRs were not statistically significant for women who reported experiencing racism in 2 or 3 domains vs none; they were 1.28 (95% CI, 0.96-1.73) (Table 4) for breast cancer mortality and 1.11 (95% CI, 0.90-1.37) (eTable 4 in Supplement 1) for all-cause mortality.

**Table 5.** Associations of Experiences With Daily and Institutional Interpersonal Racism With Breast Cancer–Specific Mortality in the Black Women’s Health Study

Experiences of racism	Deaths, No.	Person-years, No.	HR (95% CI)^a^		
			Age-adjusted^b^	Age, stage, ER status^c^	Fully adjusted^d^
Daily racism, quantile (n = 2139)					
1 (Lowest)	69	5939	1 [Reference]	1 [Reference]	1 [Reference]
2	78	6309	1.05 (0.76-1.46)	1.03 (0.74-1.43)	1.12 (0.80-1.57)
3	77	6652	0.91 (0.66-1.27)	0.93 (0.67-1.29)	1.00 (0.72-1.40)
4 (Highest)	62	4672	1.04 (0.74-1.48)	1.03 (0.72-1.45)	1.11 (0.78-1.59)
* P* for trend	NA	NA	.93	.91	.76
Institutional racism (n = 2080)					
No to all	80	7387	1 [Reference]	1 [Reference]	1 [Reference]
Yes to 1	83	7096	1.05 (0.78-1.43)	1.11 (0.82-1.51)	1.12 (0.82-1.54)
Yes to ≥2	111	8393	1.20 (0.90-1.59)	1.26 (0.95-1.68)	1.28 (0.96-1.73)
* P* for trend	NA	NA	.22	.11	.10

Abbreviations: ER, estrogen receptor; HR, hazard ratio; NA, not applicable.

^a^ *P* values for all HRs were >.05.

^b^ HRs adjusted for age at diagnosis.

^c^ HRs additionally adjusted for stage at diagnosis and ER status.

^d^ HRs additionally adjusted for tumor grade, body mass index, radiation and endocrine therapy, chemotherapy, diabetes, menopause status, alcohol intake, cigarette smoking, vigorous physical activity, education, insurance status, and region.

Lastly, in a joint analysis of self-reported daily experiences of racism and nDIS, the HRs were 1.28 (95% CI, 0.92-1.80) for women who scored high on nDIS and daily racism and 1.27 (95% CI, 0.91-1.78) for women who scored high on nDIS but low on daily racism compared with women who scored low on both. However, findings were not statistically significant.

## Discussion

In this nationwide cohort of Black women with invasive breast cancer, we found that living in disadvantaged neighborhoods was associated with worse breast cancer survival compared with living in the most privileged neighborhoods. Associations persisted even after adjustment for stage at diagnosis, breast cancer treatments, individual-level social drivers of health (eg, educational attainment), and health-related behaviors (eg, alcohol intake and physical activity). There was little evidence of higher mortality among women who reported more frequent exposure to racism in daily interactions or institutional spheres compared with none. In addition, the association between neighborhood disadvantage and breast cancer mortality was similar regardless of the frequency of personal experience of racism.

Most prior studies that assessed neighborhood concentrated advantage and/or ICE in relation to breast cancer mortality were conducted using data from majority White populations. In these studies, neighborhood disadvantage was associated with an increase in breast cancer mortality, with HRs ranging from 1.15 to 2.22.^10,15,17,32,33^ Perhaps of most interest with regard to the present study are recent findings from 2 studies based on data from the state of Georgia, each of which included large populations of both non-Hispanic White women and non-Hispanic Black women.^33,34^ Barber et al^34^ reported an increase in breast cancer mortality among White women (HR, 1.47) for the most disadvantaged vs least disadvantaged category but no evidence of an association among Black women. Luningham et al^33^ examined all-cause mortality as an outcome and found an increase among White women (HR, 1.33) but no association among Black women. The authors provided a few possible reasons, including that the chosen metrics may not fully capture the neighborhood-related factors that are most relevant to Black women. Indeed, those 2 studies used neighborhood indices based on residential data from both Black and White women. The score used in our study, which found a positive association of neighborhood disadvantage with breast cancer mortality in Black women, was based solely on residential addresses among Black women. It is possible that neighborhood-level indices and factors vary by race and ethnicity and that use of indices taken from the overall population, which are heavily weighted by White individuals, may not be appropriate for Black populations.

Regarding nSES and breast cancer mortality, there have been at least 10 prior studies among Black women.^9,11,12,13,14,16,17,32,35^ Eight of the prior studies indicated that nSES was associated with higher breast cancer-specific mortality (1.16 to 2.10),^11,12,13,14,16,32,35^ while 2 prior studies reported null results.^9,17^ A study of Asian American women also linked low neighborhood socioeconomic status to increased breast cancer mortality.^36^ Interestingly, age-adjusted findings from the present study suggested a positive association, but there was little evidence of an association after robust covariate adjustment.

To our knowledge, there have not been other publications on experiences of racism in interpersonal and institutional spheres in relation to breast cancer mortality. However, previous research has suggested that weathering^19^ may increase allostatic load,^20^ and allostatic load has been associated with higher tumor grade and larger tumor size at diagnosis.^37^ We did not observe statistically significant associations between self-reported experiences of racism and breast cancer survival; however, our racism questions were based on answers from only one time point.

### Strengths and Limitations

Our study had notable strengths. Because participant addresses, and thus neighborhood indices, were updated biennially, we could use the neighborhood index value closest to the time of diagnosis. Second, the indices were calculated based on 59 000 Black women from across the United States, maximizing relevance to our study population. Third, to our knowledge, this is the first nationwide sample of Black breast cancer survivors with data on personal experiences of racism.

Our study also had limitations. Treatment data were not complete; while we were able to control for initial treatments, we could not adjust for duration of use of endocrine therapy. Second, racism measures were taken from the time closest to breast cancer diagnosis and may not adequately capture chronic exposure to racism over a lifetime, possibly contributing to the null findings. Additionally, Black women who reported the lowest daily experiences of racism or who responded none to experiences of racism in institutional spheres are likely different (more exposed to racism) than women in other racial and ethnic groups who report none; the lack of a true no exposure or very low exposure group may bias results toward the null. However, prior research from the BWHS has found statistically significant associations between racism and various health outcomes.^38,39,40,41,42,43^ Third, we did not have access to data on the quality of treatment facility, which can impact a patient’s quality of care, nor were we able to account for insurance type. Furthermore, our study population may not be generalizable to the entire US population of Black women, as BWHS participants have higher levels of education than the general population of US Black women and have been engaged in a health research study for many years.

## Conclusions

In this study of US Black women, those who lived in neighborhoods with more concentrated disadvantage were estimated to be more likely to die from their breast cancer, even after accounting for stage at diagnosis, breast cancer treatments, individual-level lifestyle factors, and social drivers of health. The findings suggest that attributes of the neighborhood environment may contribute to poorer survival. These attributes include chronic exposure to stressors (eg, noise,^44^ lack of green space^45^), longer distance to treatment facilities,^46,47^ and limited access to a nutritional food environment.^7^ Reinvesting in communities may improve breast cancer outcomes.

## Notes

Supplement 1. **eFigure 1.** Breast Cancer Survival Analysis, Participant Inclusion Flowchart **eFigure 2.** Kaplan-Meier Curves of Neighborhood Concentrated Disadvantage and the Index of the Concentration at the Extremes by Stage at Diagnosis **eTable 1.** Associations Between Neighborhood Socioeconomic Status and Breast Cancer Mortality in the Black Women’s Health Study **eTable 2.** Associations Between Individual Components of the Neighborhood Disadvantage Score and Breast Cancer–Specific Mortality in the Black Women’s Health Study **eTable 3.** Associations Between Neighborhood-Level Indices and All-Cause Mortality in the Black Women’s Health Study **eTable 4.** Associations of Experiences With Daily and Institutional Racism With All-Cause Mortality in the Black Women’s Health Study **eTable 5.** Associations of the Joint Effects of Experiences of Neighborhood Concentrated Disadvantage and Daily Racism With Breast Cancer–Specific Mortality in the Black Women’s Health Study

Supplement 2.
Data Sharing Statement
