# Supplementary material for: Neighborhood Disadvantage, Individual Experiences of Racism, and Breast Cancer Survival
Source: JAMA Netw Open. 2025 Apr 7;8(4):e253807. doi: 10.1001/jamanetworkopen.2025.3807 (PMC11976487; doi:10.1001/jamanetworkopen.2025.3807)
Supplement: Supplement 1. — eFigure 1. Breast Cancer Survival Analysis, Participant Inclusion Flowchart eFigure 2. Kaplan-Meier Curves of Neighborhood Concentrated Disadvantage and the Index of the Concentration at the Extremes by Stage at Diagnosis eTable 1. Associations Between Neighborhood Socioeconomic Status and Breast Cancer Mortality in the Black Women’s Health Study eTable 2. Associations Between Individual Components of the Neighborhood Disadvantage Score and Breast Cancer–Specific Mortality in the Black Women’s Health Study eTable 3. Associations Between Neighborhood-Level Indices and All-Cause Mortality in the Black Women’s Health Study eTable 4. Associations of Experiences With Daily and Institutional Racism With All-Cause Mortality in the Black Women’s Health Study eTable 5. Associations of the Joint Effects of Experiences of Neighborhood Concentrated Disadvantage and Daily Racism With Breast Cancer–Specific Mortality in the Black Women’s Health Study [file jamanetwopen-e253807-s001.pdf]

Supplemental Online Content

Holder EX, Barnard ME, Xu NN, Barber LE, Palmer JR. Neighborhood disadvantage, individual experiences of racism, and breast cancer survival. *JAMA Netw Open.* 2025;8(4):e253807. doi:10.1001/jamanetworkopen.2025.3807

eFigure 1. Breast Cancer Survival Analysis, Participant Inclusion Flowchart

eFigure 2. Kaplan-Meier Curves of Neighborhood Concentrated Disadvantage and the Index of the Concentration at the Extremes by Stage at Diagnosis

eTable 1. Associations Between Neighborhood Socioeconomic Status and Breast Cancer Mortality in the Black Women's Health Study

eTable 2. Associations Between Individual Components of the Neighborhood Disadvantage Score and Breast Cancer–Specific Mortality in the Black Women's Health Study

eTable 3. Associations Between Neighborhood-Level Indices and All-Cause Mortality in the Black Women's Health Study

eTable 4. Associations of Experiences With Daily and Institutional Racism With All-Cause Mortality in the Black Women's Health Study

eTable 5. Associations of the Joint Effects of Experiences of Neighborhood Concentrated Disadvantage and Daily Racism With Breast Cancer–Specific Mortality in the Black Women's Health Study

This supplemental material has been provided by the authors to give readers additional information about their work.

© 2025 Holder EX et al. *JAMA Network Open*.

eFigure 1. Breast Cancer Survival Analysis, Participant Inclusion Flowchart

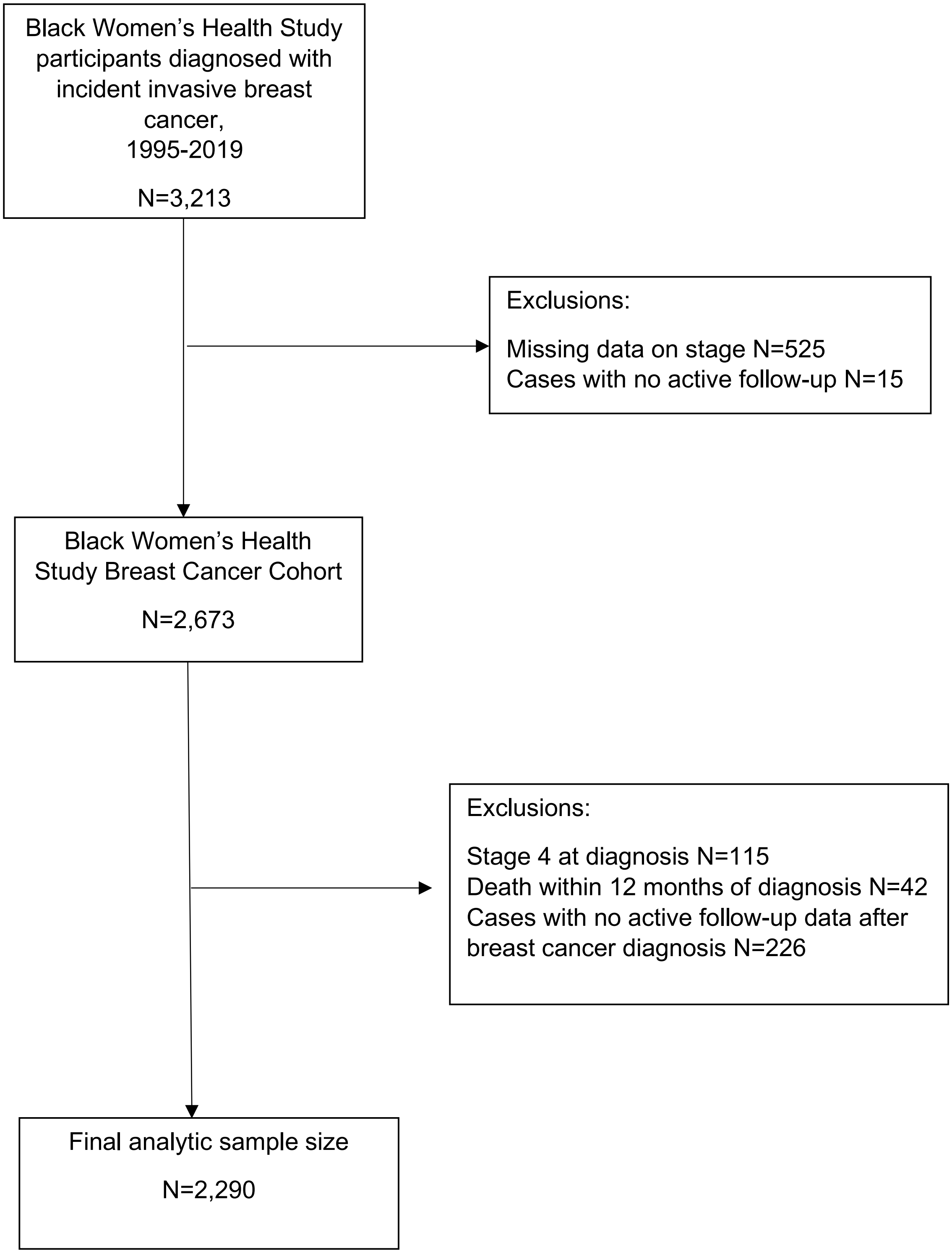

© 2025 Holder EX et al. *JAMA Network Open*.

eFigure 2. Kaplan-Meier Curves of Neighborhood Concentrated Disadvantage and the Index of the Concentration at the Extremes by Stage at Diagnosis

Stage 1

Neighborhood concentrated disadvantage

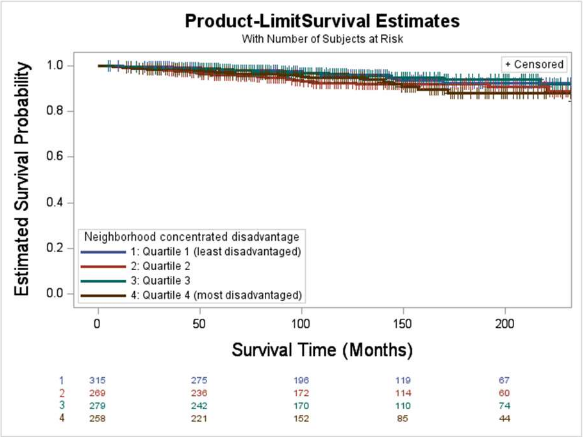

Index of the Concentration at the Extremes

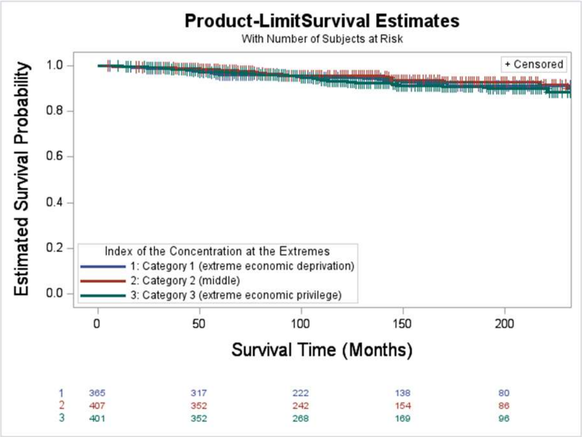

Neighborhood Socioeconomic Status

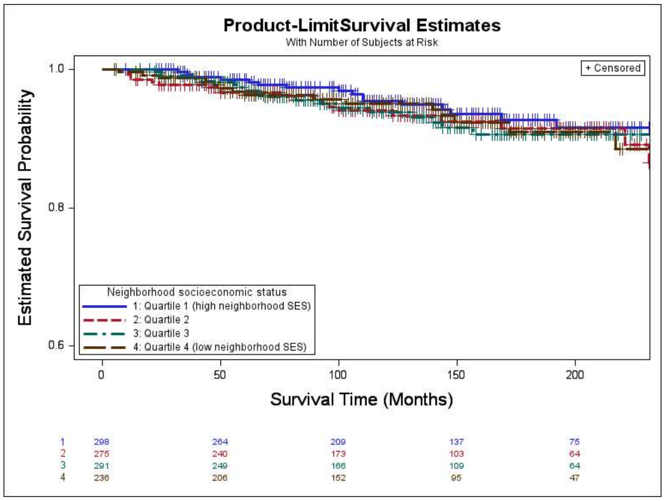

© 2025 Holder EX et al. *JAMA Network Open*.

Stage 2

Neighborhood Concentrated Disadvantage

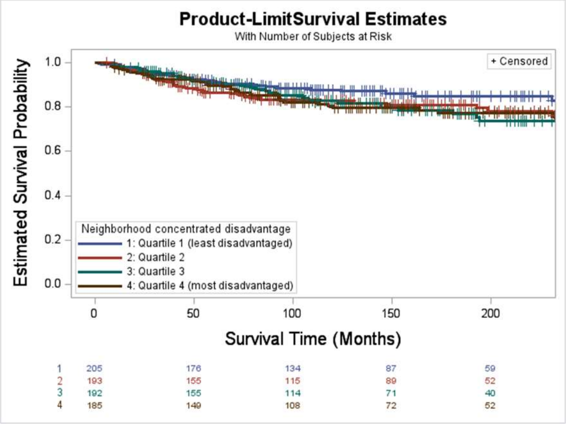

Index of Concentration at the Extremes

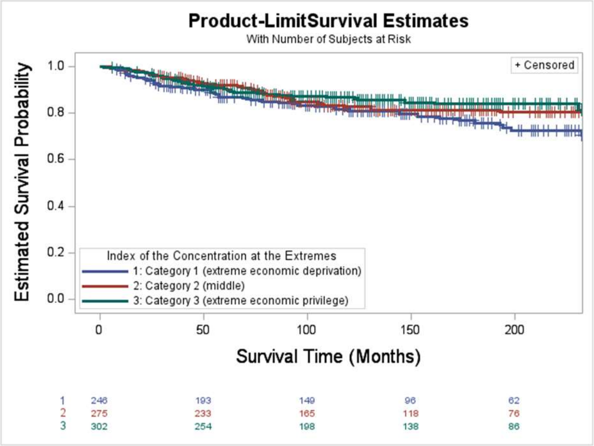

Neighborhood Socioeconomic Status

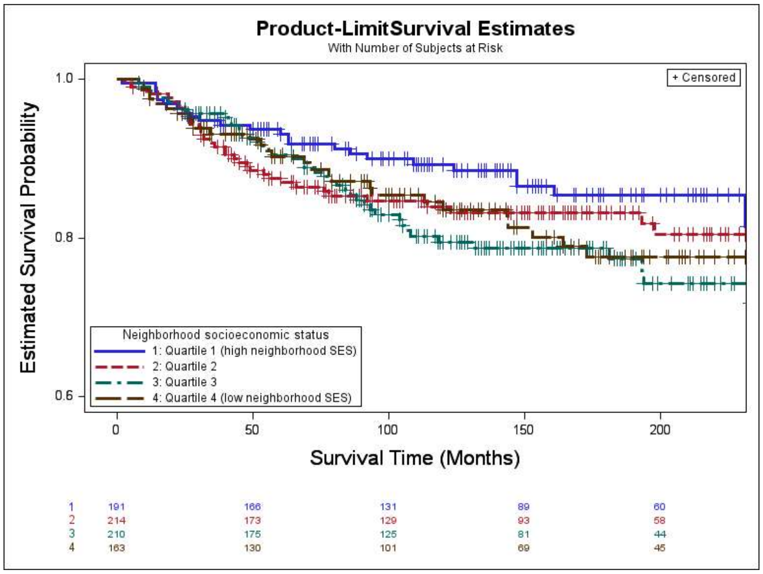

© 2025 Holder EX et al. *JAMA Network Open*.

Stage 3

Neighborhood Concentrated Disadvantage

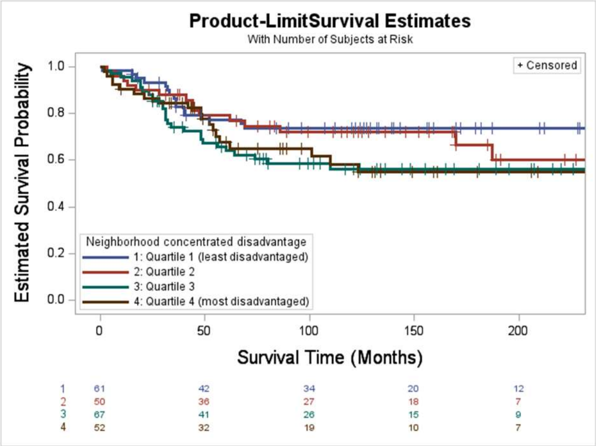

Index of Concentration at the Extremes

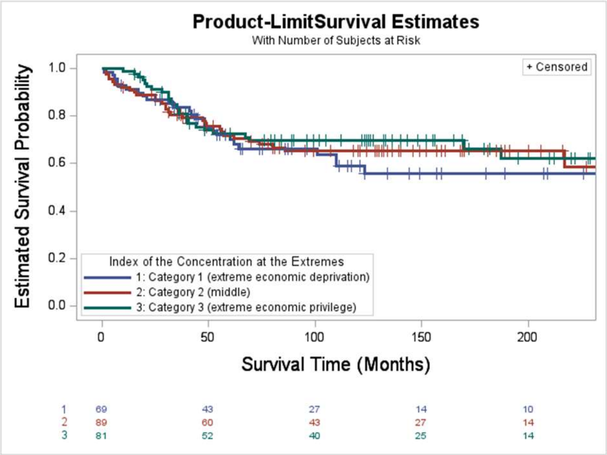

Neighborhood Socioeconomic Status

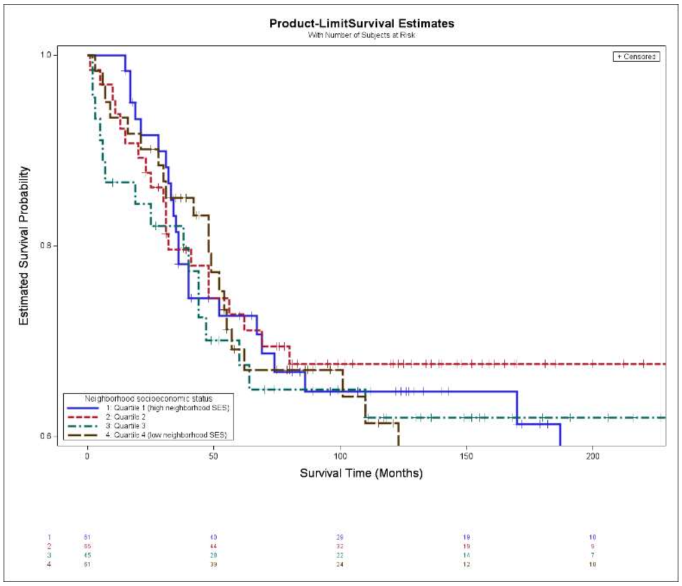

© 2025 Holder EX et al. *JAMA Network Open*.

eTable 1. Associations Between Neighborhood Socioeconomic Status (SES) and Breast Cancer Mortality in the Black Women's Health Study

Neighborhood SES (N=2,110)	Deaths	Person-years	Age-adjusted, HR* (95% CI)[1]	+ stage, ER status HR* (95% CI)[2]	Fully adjusted HR* (95% CI)[3]
Quartile 1 (low)	67	5,038	1.33 (0.94, 1.88)	1.21 (0.86, 1.70)	1.07 (0.74, 1.55)
Quartile 2	79	5,935	1.33 (0.96, 1.85)	1.31 (0.94, 1.82)	1.25 (0.88, 1.77)
Quartile 3	76	6,183	1.20 (0.86, 1.68)	1.12 (0.80, 1.56)	1.06 (0.75, 1.49)
Quartile 4 (high)	64	6,592	1.00 (ref)	1.00 (ref)	1.00 (ref)
P-trend			0.008	0.03	0.21

Abbreviations: HR, hazard ratio; CI, confidence interval; ER, estrogen receptor; SES, socioeconomic status
[1]Hazard ratios adjusted for age at diagnosis
[2]Hazard ratios additionally adjusted for stage at diagnosis, and estrogen receptor status
[3]Hazard ratios additionally adjusted for grade, body mass index, radiation and endocrine therapy, chemotherapy, diabetes, menopause status, alcohol intake, cigarette smoking, vigorous physical activity, education, insurance status, region
*P-values for all HRs were > 0.05

© 2025 Holder EX et al. *JAMA Network Open*.

eTable 2. Associations Between Individual Components of the Neighborhood Disadvantage Score and Breast Cancer–Specific Mortality in the Black Women's Health Study

Neighborhood concentrated disadvantage	Deaths	HR per standard deviation of nDIS score (95% CI)[1]
Percentage of individuals below 18 years of age	291	0.98 (0.89, 1.08)
Percentage of individuals on public assistance	291	1.05 (0.93, 1.18)
Percentage unemployed	291	1.12 (1.01, 1.24)
Percentage of Black residents	291	1.11 (0.99, 1.25)
Percentage of female-headed households	291	1.21 (1.08, 1.35)*
Percent of individuals below the poverty line	291	1.06 (0.95, 1.19)

Abbreviations: BWHS, Black Women's Health Study; HR, hazard ratio; nDIS, neighborhood concentrated disadvantage; CI, confidence interval
[1]Hazard ratios adjusted for age and stage at diagnosis, and estrogen receptor status
*Indicates $p<0.05$

© 2025 Holder EX et al. *JAMA Network Open*.

eTable 3. Associations Between Neighborhood-Level Indices and All-Cause Mortality in the Black Women's Health Study

Neighborhood-level indices	Deaths	Person-years	Age-adjusted, HR* (95% CI)[1]	+ stage, ER status HR* (95% CI)[2]	Fully adjusted HR* (95% CI)[3]
**Neighborhood concentrated disadvantage (N=2,216)**					
Quartile 1 (least disadvantaged)	128	6,574	1.00 (ref)	1.00 (ref)	1.00 (ref)
Quartile 2	121	5,923	1.09 (0.85, 1.39)	1.09 (0.85, 1.40)	1.02 (0.79, 1.32)
Quartile 3	145	5,841	1.26 (0.99, 1.59)	1.23 (0.97, 1.56)	1.07 (0.84, 1.37)
Quartile 4 (most disadvantaged)	159	5,189	1.53 (1.21, 1.93)*	1.53 (1.21, 1.93)*	1.28 (1.00, 1.64)*
P-trend			<.001	<.001	0.009
**Index of Concentration at the Extremes (N=2,235)**					
Extreme economic privilege	179	9,122	1.00 (ref)	1.00 (ref)	1.00 (ref)
Middle	190	8,620	1.07 (0.88, 1.32)	1.04 (0.85, 1.28)	0.99 (0.81, 1.22)
Extreme economic deprivation	221	7,326	1.44 (1.18, 1.75)*	1.40 (1.14, 1.70)*	1.21 (0.97, 1.49)
P-trend			<.001	<.001	0.02
**Neighborhood SES (N=2,235)**					
Quartile 1 (low)	143	5,038	1.50 (1.18, 1.91)*	1.41 (1.10, 1.79)*	1.16 (0.90, 1.51)
Quartile 2	160	5,935	1.43 (1.13, 1.81)*	1.45 (1.15, 1.84)*	1.27 (0.99, 1.62)
Quartile 3	136	6,183	1.17 (0.92, 1.50)	1.13 (0.88, 1.44)	1.06 (0.82, 1.36)
Quartile 4 (high)	121	6,592	1.00 (ref)	1.00 (ref)	1.00 (ref)
P-trend			<.001	<.001	0.03

Abbreviations: HR, hazard ratio; CI, confidence interval; ER, estrogen receptor; SES, socioeconomic status
[1]Hazard ratios adjusted for age at diagnosis
[2]Hazard ratios additionally adjusted for stage at diagnosis, and estrogen receptor status
[3]Hazard ratios additionally adjusted for grade, body mass index, radiation and endocrine therapy, chemotherapy, diabetes, menopause status, alcohol intake, cigarette smoking, vigorous physical activity, education, insurance status, region
*P-values for all HRs were > 0.05

© 2025 Holder EX et al. *JAMA Network Open*.

eTable 4. Associations of Experiences With Daily and Institutional Racism With All-Cause Mortality in the Black Women's Health Study

	Deaths	Person-years	Age-adjusted, HR* (95% CI)[1]	+ stage, ER status HR* (95% CI)[2]	Fully adjusted HR* (95% CI)[3]
**Daily Racism (N=2,139)**					
Quantile 1 (lowest)	146	5,939	1.00 (ref)	1.00 (ref)	1.00 (ref)
Quantile 2	146	6,309	1.07 (0.85, 1.35)	1.06 (0.84, 1.34)	1.06 (0.84, 1.35)
Quantile 3	154	6,652	1.05 (0.83, 1.32)	1.05 (0.83, 1.32)	1.05 (0.83, 1.33)
Quantile 4 (highest)	110	4,672	1.11 (0.86, 1.43)	1.08 (0.84, 1.39)	1.09 (0.85, 1.42)
P-trend			0.48		0.53
**Institutional Racism (N=2,080)**					
No to all	165	7,387	1.00 (ref)	1.00 (ref)	1.00 (ref)
Yes to 1	163	7,096	1.02 (0.82, 1.26)	1.04 (0.84, 1.30)	1.07 (0.85, 1.33)
Yes to ≥2	200	8,393	1.07 (0.87, 1.32)	1.10 (0.89, 1.35)	1.11 (0.90, 1.37)
P-trend			0.50	0.39	0.35

Abbreviations: HR, hazard ratio; CI, confidence interval; ER, estrogen receptor
[1]Hazard ratios adjusted for age at diagnosis
[2]Hazard ratios additionally adjusted for stage at diagnosis, and estrogen receptor status
[3]Hazard ratios additionally adjusted for grade, body mass index, radiation and endocrine therapy, chemotherapy, diabetes, menopause status, alcohol intake, cigarette smoking, vigorous physical activity, education, insurance status, region
*P-values for all HRs were > 0.05

© 2025 Holder EX et al. *JAMA Network Open*.

eTable 5. Associations[1] of the Joint Effects of Experiences of Neighborhood Concentrated Disadvantage and Daily Racism With Breast Cancer–Specific Mortality in the Black Women's Health Study

Daily Racism x nDIS (N=1,991)	Deaths	HR* (95% CI)
low racism/ low nDIS	65	1.00 (ref)
high racism/ low nDIS	56	0.91 (0.64, 1.30)
low racism/ high nDIS	74	1.27 (0.91, 1.78)
high racism/ high nDIS	70	1.28 (0.92, 1.80)

Abbreviations: nDIS; neighborhood concentrated disadvantage; HR, hazard ratio; CI, confidence interval
[1]Hazard ratios adjusted for age at diagnosis, stage at diagnosis, and estrogen receptor status
*P-values for all HRs were > 0.05

© 2025 Holder EX et al. *JAMA Network Open*.
