# Supplementary material for: Neighborhood Disadvantage, Individual Experiences of Racism, and Breast Cancer Survival
Source: JAMA Netw Open. 2025 Apr 7;8(4):e253807. doi: 10.1001/jamanetworkopen.2025.3807 (PMC11976487; doi:10.1001/jamanetworkopen.2025.3807)
Supplement: Supplement 2. — Data Sharing Statement [file jamanetwopen-e253807-s002.pdf]

Data Sharing Statement

Holder. Neighborhood Disadvantage, Individual Experiences of Racism, and Breast Cancer Survival. *JAMA Netw Open*. Published April 07, 2025.
doi:10.1001/jamanetworkopen.2025.3807

Data
Data available: No
Supporting Documents
Document types: None

Additional Information
Explanation: The data generated during and/or analyzed during the current study are not publicly available due to IRB protocol but are available upon reasonable request from the study PI (jpalmer@bu.edu) and https://www.bu.edu/bwhs/for-researchers/ (for researchers).
